# Early lessons from the implementation of universal respiratory syncytial virus prophylaxis in infants with long-acting monoclonal antibodies, Galicia, Spain, September and October 2023

**DOI:** 10.2807/1560-7917.ES.2023.28.49.2300606

**Published:** 2023-12-07

**Authors:** Federico Martinón-Torres, Susana Mirás-Carballal, Carmen Durán-Parrondo

**Affiliations:** 1 Genetics, Vaccines and Paediatric Infectious Diseases Research Group (GENVIP), Instituto de Investigación Sanitaria de Santiago (IDIS), Santiago de Compostela, Galicia, Spain; 2 Translational Paediatrics and Infectious Diseases, Hospital Clínico Universitario and University of Santiago de Compostela (USC), Santiago de Compostela, Spain; 3 World Health Organization Collaborating Centre for Vaccine Safety, Santiago de Compostela, Spain; 4 Centro de Investigación Biomédica en Red de Enfermedades Respiratorias (CIBERES), Instituto de Salud Carlos III, Madrid, Spain; 5 Dirección Xeral de Saude Pública, Consellería de Sanidade, Xunta de Galicia, Galicia, Spain

**Keywords:** Respiratory Syncytial Virus, nirsevimab, implementation, immunoprophylaxis, long-acting monoclonal antibodies

## Abstract

A monoclonal antibody for universal respiratory syncytial virus prophylaxis in infants has recently been licensed. We share our experiences of integrating nirsevimab into the regional immunisation programme in Galicia, Spain. After a 3-week hospital-based immunisation campaign with flexible individualised appointments and educational activities, nirsevimab uptake was 97.5% in the high-risk group, 81.4% in the catch-up group and 92.6% in infants born during the campaign. This successful implementation strategy can serve as a model and may inform other countries’ programmatic deliberations.

The respiratory syncytial virus (RSV) causes a substantial disease burden worldwide and specifically in all infants, including healthy ones [1]. After more than six decades of research, two immunisation products that can protect the broad infant population were licensed in 2023: a long-acting monoclonal antibody (nirsevimab, Beyfortus, AstraZeneca/Sanofi Pasteur) and a maternal vaccine (inactivated pre-F RSVA/RSVB vaccine, Abrysvo, Pfizer) [2]. In March 2023, Galicia (Spain) introduced universal RSV prophylaxis in infants into its national immunisation programme, using nirsevimab [3]. The early experience of its practical implementation, reported here, may inform other countries’ programmatic deliberations.

## The campaign design

The decision to start RSV universal prophylaxis in the public-funded immunisation programme of Galicia was based on careful examination of the burden of disease in the region and the potential impact and efficiency of the measure [1,4-8]. At the moment of the decision, in 2023, nirsevimab was the only option available in Spain as the maternal vaccine had not been approved yet.

The 2023/24 immunisation campaign against RSV in Galicia with nirsevimab started on 25 September 2023 and will conclude on 31 March 2024, when the RSV season is expected to end based on data of the previous 12 seasons (excluding the 2020/21 and 2021/22 seasons, which took place during the COVID-19 pandemic). It is structured around three immunisation groups: seasonal, catch-up and high-risk, which are defined in detail in the Box.

Box **Groups eligible for the respiratory syncytial virus prophylaxis campaign with nirsevimab in Galicia, Spain, 2023/24**

**Seasonal**:

Any infant born during the RSV season, i.e. from 25 September 2023 to 31 March 2024, will receive one dose of nirsevimab in the hospital, in the first 24 h of life, unless medically contraindicated.

**Catch-up:**

Any infant 0–6 months-old at the start of the RSV season, i.e. born between 1 April and 24 September 2023, will receive one dose of nirsevimab in their reference hospital, following a flexible electronic personal appointment, within 3 weeks of the start of the RSV prophylaxis campaign^a^.

**High-risk:**

Any child 6–24 months of age at the start of the RSV season, with any of the risk conditions of severe RSV disease listed below, will be offered an appointment in the first week of the campaign to receive nirsevimab in their reference hospital^a^.

- congenital heart diseases with considerable haemodynamic impact, whether cyanotic or acyanotic,
- bronchopulmonary dysplasia,
- severe immunosuppression: oncohaematological diseases, continuous treatment with immunosuppressants or primary immunodeficiencies (with special attention to severe combined immunodeficiency and congenital agammaglobulinaemia),
- congenital metabolic disorders,
- neuromuscular diseases,
- severe pulmonary diseases,
- genetic syndromes causing considerable respiratory problems,
- trisomy 21,
- cystic fibrosis,
- patients in palliative care.

Premature infants younger than 35 weeks (including those with a gestational age < 29 weeks), a single dose before reaching 12 months of age.

RSV: respiratory syncytial virus.^a^Those who miss the original appointment for any reason will be rescheduled for a hospital appointment and, if the situation persists once the hospital-centred campaign is finished, they will be referred to their primary care paediatrician for the immunisation.Seasonality of RSV in Galicia, based on the data for the previous 12 seasons (excluding the 2020/21 and 2021/22 seasons during the COVID-19 pandemic), was determined to be between 25 September 2023 and 31 March 2024.

All groups receive a single intramuscular dose of nirsevimab of 50 or 100 mg in the thigh or the deltoid muscle, according to the subject’s weight. The campaign was preceded by informative, educational and training activities for healthcare professionals, and materials such as leaflets, posters, websites and press releases were disseminated to the general population [4]. A distinctive aspect of the campaign is that nirsevimab administration occurs through Galicias’s network of hospitals. Children in the high-risk and catch-up groups received flexible appointments that were individually scheduled electronically over a 3-week period (25–30 September for the high-risk group, and 1–15 October for the catch-up group), from 10:00 to 19:00, including weekends. Infants classified as ‘seasonal’ receive the nirsevimab dose in the hospital in the first 24 h of life, except when there is a medical contraindication. Any eligible children who missed the original hospital-based immunisation appointment are actively rescheduled and recaptured through the network of primary care centres. 

Surveillance of severe adverse events following immunisation is routinely performed in all subjects through the Galician pharmacovigilance system. In addition, active surveillance of any potential adverse event or hospitalisation in the first 3 weeks since nirsevimab administration is conducted in the preterm population.

## Early results of the hospital-based active catch-up campaign

After the 3-week hospital-based active immunisation campaign, nirsevimab uptake was 97.5% (317/325) in the high-risk group and 81.4% (5,820/7,150) in the catch-up group. The immunisation uptake in the infants born since the start of the campaign had reached 92.6% (1,104/1,192) by 31 October. No severe adverse effects have been reported so far after 7,241 administered doses.

## The rationale of the campaign

The use of the hospital network as the campaign’s logistical foundation aimed to achieve a rapid and high vaccine uptake before the onset of RSV circulation. This decision was based on the success of a previous experience with this same approach in the universal influenza vaccination campaign in children in the 2021/22 season [9]. All eligible children receive an individualised and flexible electronic appointment to be immunised in their reference hospital. This active hospital-based campaign runs for 3 weeks, including weekends. After that period, any remaining infant and child targeted for immunisation is recaptured through the network of primary care paediatricians.

For those born during RSV season, early nirsevimab administration is crucial to minimise newborn’s spontaneous contact with RSV during their most vulnerable period. Administering it within the first 24 h of birth reduces this exposure risk considerably. For this reason, hospitals were selected as the location of choice to perform the immunisation. Waiting until the first scheduled visit with the primary care paediatrician to administer nirsevimab may expose the infant unnecessarily during the interval after hospital discharge.

In Galicia, vaccine hesitancy and declining coverages are not predominant issues, but the introduction of a universally administered monoclonal antibody represented a novel challenge. This initiative was anticipated to potentially elicit reservations from both parents and healthcare providers, due to its unprecedented nature, therefore, a key aspect of our campaign was to proactively address and mitigate any a priori doubts regarding this innovative approach. The roll-out of the campaign was preceded by informative and educational meetings for the different healthcare professionals involved in the infants’ care and/or immunisation practices. The official instruction driving the campaign was widely distributed. Specific materials, including webinars, brief video-clips and leaflets with questions and answers were made available through distribution lists and the public health webpage [10]. Press releases and media engagement were coupled with the other informative and educational efforts to reach the parents of the target population. 

The effectiveness and impact of the campaign will be evaluated by the NIRSE-GAL study (www.nirsegal.es), approved by the reference ethics committee (CEIC 2023–377). The primary objective of the study is to evaluate the effectiveness of nirsevimab on hospitalisation for RSV in the different target groups, using as background reference the RSV hospitalisation data for the period 2016 to 2023. In addition, the impact of nirsevimab on primary care/healthcare consumption and its longitudinal impact on wheezing and asthma will be also assessed.

The novelty of nirsevimab presented challenges in finding extensive cost-effectiveness studies, making the initial approach of Galician authorities a pragmatic public health decision aimed at addressing immediate healthcare needs based on the burden of disease and the cost savings from limiting palivizumab consumption which is still used widely elsewhere as prevention of RSV infections in high-risk infants. The price of nirsevimab in Galicia has been set for only one year, and a reassessment of the pricing and cost-effectiveness will be warranted considering the actual savings accrued from reduced hospitalisations and medical interventions measured through the NIRSE-GAL study.

## Conclusions

We believe that the success in the implementation phase of nirsevimab in Galicia is based on a robust information and education campaign and a hospital-based roll-out, enabling rapid immunisation of the target population before the start of RSV circulation. Reducing the RSV burden is a public health priority. In addition to long-acting monoclonal antibodies such as nirsevimab, a safe and efficacious inactivated vaccine has also been approved for use in pregnant women, providing an alternative or complementary option for passive RSV prophylaxis in the newborn. Several countries are currently considering universal RSV prophylaxis in infants with the currently available maternal vaccine or long-acting monoclonal antibodies. Such an undertaking requires judicious yet swift action. Galicia's experience implementing nirsevimab may aid policymakers in developing an RSV immunisation prevention strategy.
